# Prognostic Significance of SOCS3 in Patients With Solid Tumors: A Meta-Analysis

**DOI:** 10.3389/fsurg.2021.802143

**Published:** 2022-02-28

**Authors:** Xia Zheng, Jie Shao, Sihui Wei, Yu Gu, Jun Qian

**Affiliations:** ^1^Oncology Department, Affiliated Hospital of Nanjing University of Chinese Medicine, Nanjing, China; ^2^Oncology Department, Jiangsu Province Hospital of Chinese Medicine, Nanjing, China; ^3^Oncology Department, Third People‘s Hospital of Province, Wuhan, China

**Keywords:** suppressor of cytokine signaling 3 (SOCS3), solid tumor, prognosis, meta-analysis, radical resection

## Abstract

**Background:**

The identification of reliable biomarkers for predicting disease recurrence and the survival of patients with cancer is of great importance. Numerous previous studies have revealed that the abnormal expression of the suppressor of cytokine signaling 3 (SOCS3) was associated with patient outcomes. However, these results were inconsistent. The aim of the present study was to assess the prognostic value of SOCS3 in patients with solid tumors.

**Methods:**

Studies focusing on the prognostic value of SOCS3 in solid tumors were searched for in the PubMed, Embase, Web of Science, and Scopus databases. We included studies that compared disease-free survival (DFS) and overall survival based on different levels of SOCS3. Other outcomes (e.g., Edmondson grading, tumor size, tumor vascular invasion, lymph node invasion, and distant metastasis) were also considered. The hazard ratio (HR)/risk ratio (RR) and corresponding 95% CI were determined.

**Results:**

Twelve studies with 1,551 patients were included in this meta-analysis. The pooled analysis demonstrated that the higher expression of SOCS3 was significantly associated with better disease-free survival (HR:0.36, 95% CI:0.17–0.77, *P* < 0.001) and overall survival (HR:0.45, 95% CI:0.32–0.62, *P* < 0.001) compared with low expression. Moreover, SOCS3 expression was closely correlated with the Edmondson grading [odds ratio (OR):0.77, 95% CI:0.61–0.98, *P* = 0.033], vascular invasion (OR:0.63, 95% CI:0.52–0.78, *P* < 0.001), and distant metastasis (OR:0.73, 95% CI:0.51–1.03, *P* = *0.0*76). However, the levels of SOCS3 were not significantly associated with tumor size (OR:0.85, 95% CI:0.71–1.03, *P* = 0.090) and lymph node invasion (OR:0.73, 95% CI:0.51–1.03, *P* = 0.076).

**Conclusion:**

Increased SOCS3 expression in tumor mass was associated with better DFS and OS, suggesting it might be a novel and reliable biomarker for predicting the risk of cancer recurrence and mortality.

## Introduction

According to the most recently published data, the global cancer-related morbidity and mortality rates in 2020 were estimatedto be 19.3 and 10.0 million, respectively. This evidence revealed that malignancy has become a major public health concern (1). Surgical resection is considered the main curative therapeutic strategy for most types of solid tumors. However, most patients with such tumors experience postoperative recurrence. Although chemotherapy, radiotherapy, and targeted therapy have made remarkable progress in cancer treatment, patients‘ survival remains limited. Immunotherapy, especially for immune checkpoint inhibitors, has been applied in a variety of tumors and prolonged patients‘ survival significantly. Immune-based combinations have been recommended as the first-line therapy in most malignancies such as hepatocellular carcinoma (HCC) (2, 3). Nonetheless, some patients did not respond to this novel treatment option with unsatisfactory survival. According to previous studies, it is necessary to identify biomarkers to predict the outcomes in patients who received immune-based therapy (4). Thus far, risk factors linked to patients‘ outcomes have been poorly understood. Therefore, the identification of hypersensitive and specific biomarkers for predicting patient outcomes is urgently warranted.

Currently, various clinicopathological factors (e.g., Edmondson grading, tumor size, lymph nodes invasion, and distant metastasis) have been recognized as common predictors of patient outcome. Accordingly, multiple tumor staging systems (e.g., TNM) have been developed and applied to the management of cancer in clinical practice. However, the accuracy of these systems remains unsatisfactory.

Owing to their inhibitory effect on multiple cytokine-related signaling pathways, members of the suppressor of cytokine signaling (SOCS) protein family are considered potential prognostic molecules in patients with cancer. Particularly, it has been found that the SOCS 3 (SOCS3) expression is lower in tumor tissues compared with adjacent tissues; this difference in expression may influence patient outcomes (5, 6). Interestingly, SOCS3 acts as a double-edged sword in the regulation of cancer biology. For example, a recent study suggested that SOCS3 inhibited the proliferation of breast cancer cells *in vitro* (7). However, another study demonstrated that SOCS3 mediated interferon-α resistance in renal cell carcinoma (8).

Although SOCS3 methylation has been demonstrated as a reliable prognostic factor in HBV infection-related HCC cases (9), previous clinical studies have yielded inconsistent data regarding the prognostic significance of SOCS3. Multiple retrospective cohort studies revealed that elevated SOCS3 expression was correlated with favorable disease-free survival (DFS) and overall survival (OS) in patients with HCC (10, 11), colorectal cancer (12), gastric cancer (13–15), breast cancer (16), cholangiocarcinoma (17), ovarian cancer (18), and prostatic cancer (19). However, Jiang et al. (20). have demonstrated that the expression of SOCS3 cannot predict the postoperative risk of tumor recurrence in patients with HCC. Conversely, a study reported by Bekki et al. (21) suggested that higher levels of SOCS3 were associated with an increased risk of recurrence in undifferentiated pleomorphic sarcoma.

Therefore, we performed a meta-analysis of data collected from published research studies to re-assess the prognostic value of SOCS3 in patients with solid tumors.

## Methods

### Search Strategy

Published studies potentially related to solid tumors and SOCS3 expression were extracted from the PubMed, Embase, Scopus, and Web of Science databases in October 2020. The keywords “cancer,” “carcinoma,” “suppressor of cytokine signaling 3,” and “prognosis,” as well as related abbreviations, were used for the screening and identification of candidate studies to be included in the meta-analysis. Multiple synonyms were also utilized.

### Inclusion and Exclusion Criteria

Eligible studies were identified using the following criteria: (1) research addressing the relationship between the outcomes of patients with solid tumors and SOCS3 expression, (2) detection of SOCS3 expression in tumor tissues using immunohistochemistry, and (3) confirmation of all solid tumors through pathological examination.

The exclusion criteria for this meta-analysis were: (1) other types of publications (i.e., reviews, conference abstracts, case reports, or comments); (2) *in vivo* or *in vitro* research studies; (3) lack of data on DFS or OS; (4) use of data from public databases; and (5) lack of hazard ratios (HRs) and 95% confidence intervals (CIs) as effective measurements.

### Data Management and Outcome Assessment

Using the aforementioned criteria, available articles were independently selected and reviewed by two investigators through abstract and full-text reading. In case of disagreement between the investigators, a consensus was reached through discussion with a senior investigator. The HRs and 95% CIs of OS and DFS were collected and recognized as effective measurements. Univariate and multivariate analyses were performed, and the studies or data with more accurate HRs and 95% CIs were subsequently selected for the meta-analysis.

### Quality Assessment

The UK Cochrane Center of Evidence (2009) was used to estimate the level of evidence in the studies. The quality of the retrospective cohort studies was assessed using the Newcastle–Ottawa scale (22). This scale consists of three factors, namely the selection of patients, comparability of the study groups, and assessment of outcome. The maximum total score is nine; scores ≥6 denoted high-quality studies and were also a pre-setting selection criterion in this report.

### Statistical Analysis

Hazard ratios (HRs) and the corresponding 95% CIs were calculated to pool the functional outcomes. Statistical heterogeneity among the studies was assessed using chi-square tests, with *P* < 0.05 or I^2^ >50% denoting statistical significance. In the absence of evident heterogeneity, a fixed-effects model was utilized; otherwise, a random-effects model was selected to minimize the heterogeneity, followed by subgroup and sensitivity analyses. Funnel plots, Egger's test, and Begg's test were used to examine publication bias. All statistical analyses were performed using the STATA version 14.0 (StataCorp, College Station, TX, USA) software.

## Results

### Characteristics of Selected Articles

**Figure 1:**
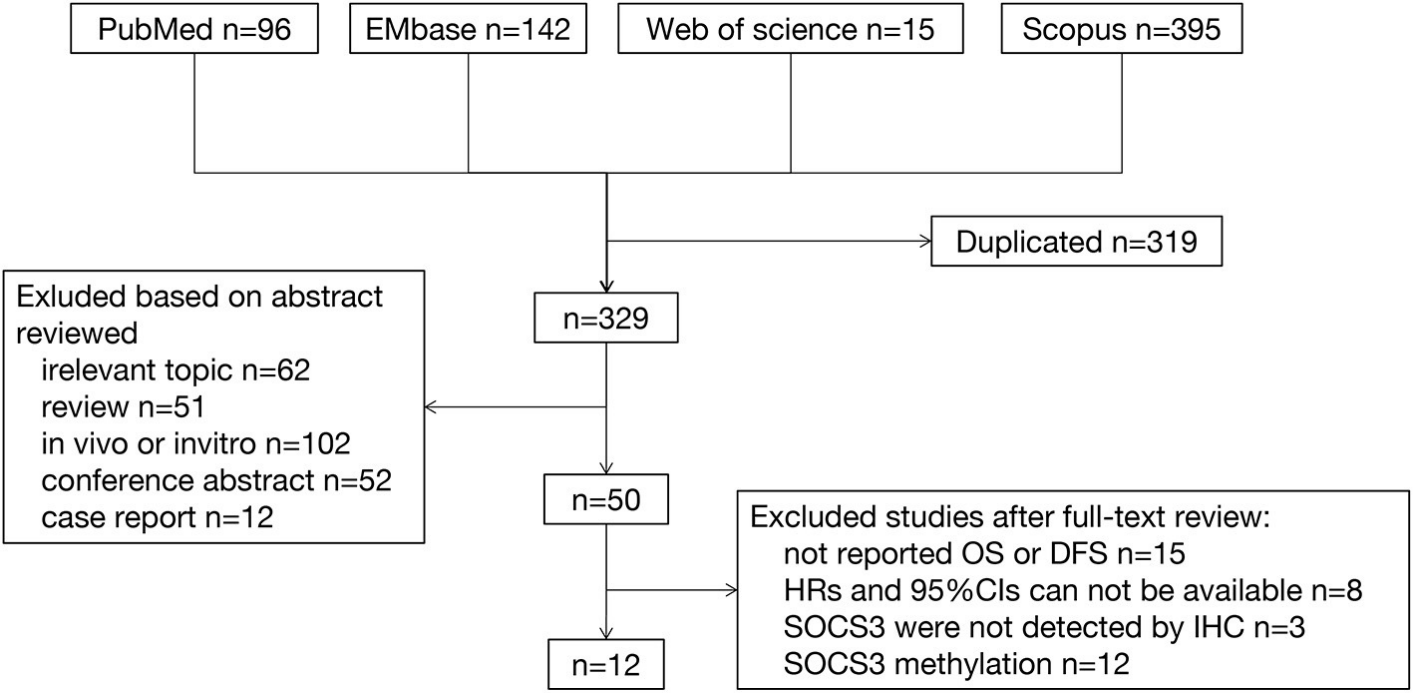
Flow of studies selection.

After removing duplicated publications (*n* = 319), 329 articles were selected for screening. By scanning the titles and abstracts of these articles, 279 publications were excluded; of those, 62 were unrelated, 51 were reviews, 102 described *in vivo* or *in vitro* studies, 52 were conference abstracts, and 12 were case reports. According to the aforementioned criteria, 38 studies were excluded for the following reasons: (1) lack of data on DFS and OS (*n* = 15), (2) lack of HRs and 95% CIs (*n* = 8), (3) lack of immunohistochemistry analysis for the detection of SOCS3 expression, and (4) exclusive focus on SOCS3 methylation (*n* = 12) (Figure 1).

In total, 1,551 patients from 12 retrospective cohort studies were included in the present analysis (10–21). The level of evidence was 2a. Based on the Newcastle–Ottawa Scale, all studies received a quality score of 6–9 (Table 1).

**Table 1:** Characteristics of included studies.

**References**	**Country**	**Mean age (year)**	**n (male%)**	**Cancer type**	**Stage**	**Treatment options**	**Follow-up time (month)**	**Analysis variate**	**Outcomes**	**Level of evidence**	**NOS**
Chen et al. (10)	China	na	74 (91.9%)	HCC	pT1-3N0M0 (8th)	Surgery	60.0	M	OS	2a	8
Bekki et al. (21)	Japan	na	75 (40%)	UPS	II-IV (7th)	na	na	M	OS	2a	7
Chu et al. (12)	China	68.72	88 (52.3%)	CRC	I-IV (7th)	Surgery	46.62	M	OS	2a	8
Deng et al. (15)	China	57.4	107 (66.45)	GC	na	Surgery	37.0	M	OS	2a	8
Jiang et al. (20)	China	51.4	176 (84.1%)	HCC	na	Surgery	56.5	M	DFS, OS	2a	9
Li et al. (14)	China	57.0	186 (68.8%)	GC	I-IV (6th)	Surgery	40.7	M	OS	2a	8
Pierconti et al. (19)	Italy	na	65 (100%)	PCa	pT2-3N0M0 (7th)	Radical prostatectomy	na	M	DFS	2a	6
Shang et al. (18)	China	54.0	136 (0%)	OC	I-IV (FIGO)	Surgery	na	M	OS	2a	6
Wang et al. (17)	China	61.1	86 (37.2%)	CCA	I-IVA (7th)	Surgery	23.0	M	OS	2a	8
Xu et al. (13)	China	48.0	105 (65.7%)	GC	I-IV (7th)	Surgery	30.0	M	OS	2a	8
Ying et al. (16)	China	52.8	367 (0%)	BC	I-III (7th)	Surgery	43.3	M	DFS	2a	7
Zhao et al. (11)	China	50.4	85 (78.8%)	HCC	na	Surgery	30.0	M	DFS, OS	2a	8

*HCC, hepatocellular carcinoma; UPS, Undifferentiated Pleomorphic Sarcoma; Pca, prostate cancer; CRC, colorectal cancer; OC, ovarian cancer; GC, gastric cancer; BC, breast cancer; CCA, cholangiocarcinoma; DFS, disease-free survival; OS, overall survival; M, multivariate analysis*.

### Predictive Role of SOCS3 in DFS and OS

**Figure 2:**
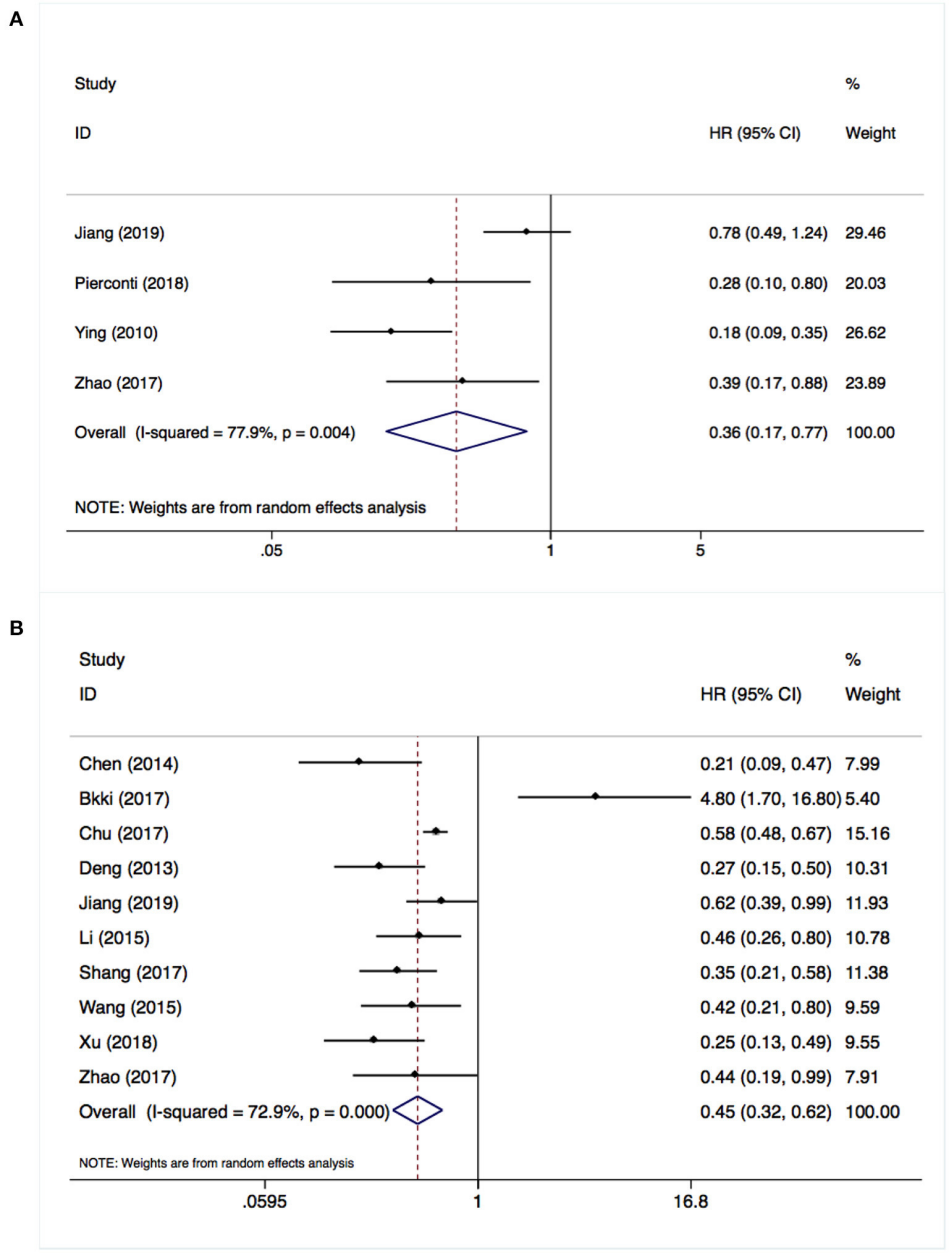
Overexpressed suppressor of cytokine signaling 3 (SOCS3) was associated with better disease-free survival **(A)** and overall survival **(B)**.

Four studies including 693 patients investigated the relationship between SOCS3 expression and the risk of tumor recurrence. Considering the degree of heterogeneity (I^2^ = 77.9%), a random-effects model was utilized for the analysis of these data. We found that an elevated SOCS3 expression in tumor tissues was significantly correlated with better DFS (HR:0.36, 95% CI:0.17–0.77, *P* < 0.001) vs. low expression (Figure 2A). Similarly, a pooled analysis of 10 studies with 1,119 cases using the random-effects model (I^2^ = 72.9%) revealed that higher levels of SOCS3 were significantly associated with better OS vs. low levels (HR:0.45, 95% CI:0.32–0.62, *P* < 0.001) (Figure 2B, Table 2).

**Table 2:** Results of meta-analysis of interested outcomes.

**Outcomes/pathological factors**	**Cohort number**	**Case number**	**HR/RR(95%CI)-Model**	***P***	**Heterogeneity**		**Public bias**	
					**I^**2**^ (%)**	***P***	**Egger test *P***	**Begg test *P***
DFS	4	693	0.36 (0.17–0.77)-random	<0.001	77.9	0.004	0.381	0.734
OS	10	1,119	0.45 (0.32–0.62)-random	<0.001	72.9	<0.001	0.437	0.371
Differentiation (III–IV vs. I–II)	8	1,118	0.77 (0.61–0.98)-random	0.033	72.9	<0.001	0.612	0.902
Tumor size (large vs. small)	7	1,183	0.85 (0.71–1.03)-random	0.090	67.2	0.006	0.110	0.230
Vascular invasion (present vs. absence)	6	876	0.63 (0.52–0.78)-fixed	<0.001	9.40	0.356	0.856	1.000
Lymph nodes metastasis (with vs. without)	8	1,252	0.73 (0.51–1.03)-random	0.076	85.0	<0.001	0.915	1.000
Distance metastasis (with vs. without)	4	557	0.56 (0.40–0.78)-fixed	<0.001	34.2	0.208	0.107	0.308

### Correlation Between SOCS3 Expression and Clinicopathological Features

**Figure 3:**
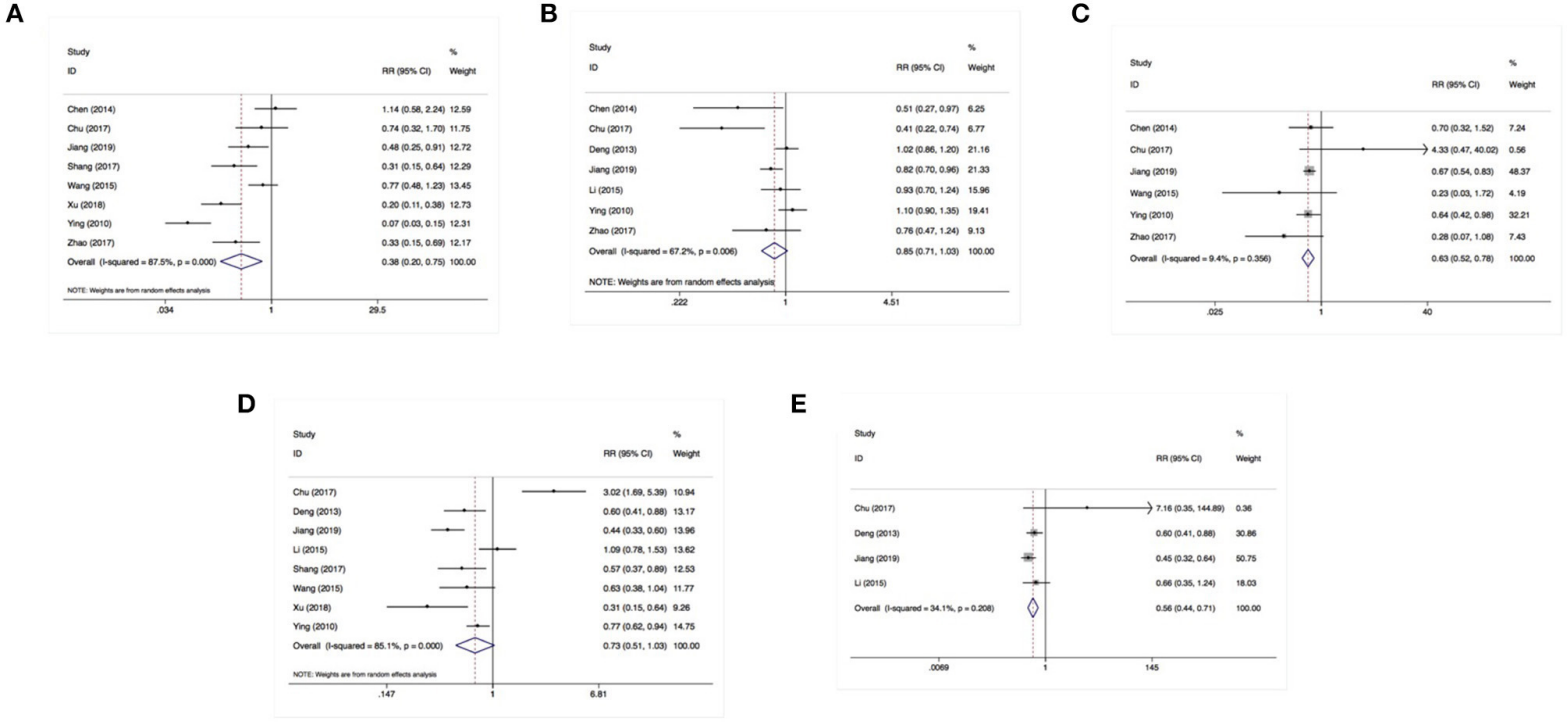
SOCS3 expression was associated with Edmondson grading **(A)**, vascular invasion **(C)**, distant metastasis **(E)** instead of tumor size **(B)** or lymph nodes invasion **(D)**.

Eight studies involving 1,118 patients focused on the relationship between SOCS3 expression and Edmondson grading. A pooled analysis with a random-effects model (I^2^ = 72.9%) revealed that the lower expression of SOCS3 was correlated with poorly differentiated tumors [risk ratio (RR):0.77, 95% CI:0.61–0.98, *P* = 0.033] (Figure 3A). However, according to the results of a meta-analysis of seven studies using a random-effects model (I^2^ = 67.2), SOCS3 expression was not associated with tumor size (RR:0.85, 95% CI:0.71–1.03, *P* = 0.090) (Figure 3B).

Using a fixed-effects model (I^2^ = 9.40%), a pooled analysis of six studies (including 876 cases) demonstrated that the lower expression of SOCS3 increased the risk of tumor vascular invasion (RR:0.63, 95% CI:0.52–0.78, *P* < 0.001) (Figure 3C). In contrast, an analysis of eight studies with a random-effects model (I^2^ = 85.0%) revealed that SOCS3 expression did not have an obvious impact on lymph node invasion (RR:0.73, 95% CI:0.51–1.03, *P* = 0.076) (Figure 3D). An analysis of four studies with a fixed-effects model showed that higher SOCS3 expression significantly reduced the risk of metastasis vs. low expression (RR:0.56, 95% CI:0.40–0.78, *P* < 0.001) (Figure 3E).

### Sensitivity, Stratification, and Bias Analysis

**Figure 4:**
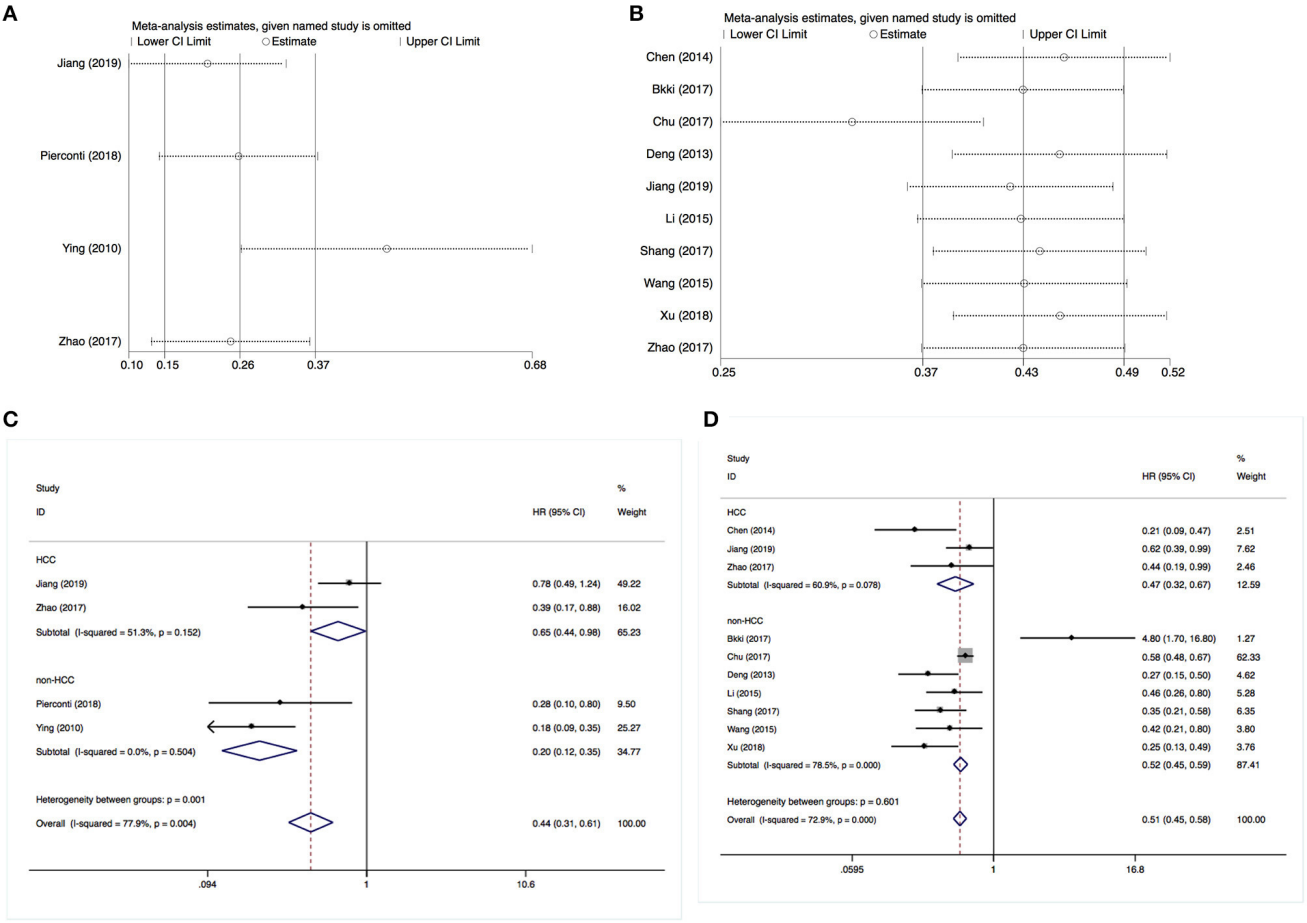
Sensitivity and stratification analysis results. **(A)** Sensitivity analysis of disease-free survival. **(B)** Sensitivity analysis of overall survival. **(C)** Stratification analysis of disease-free survival. **(D)** Stratification analysis of overall survival.

For the sensitivity analysis, we employed a leave-one-out approach to examine the stability of the pooled analysis results concerning DFS and OS. The exclusion of any single study did not alter the statistical significance of the results, indicating that the results of this meta-analysis were stable and robust (Figures 4A,B).

Subsequently, we performed a stratification analysis by the studies that enrolled HCC (*n* = 3) or non-HCC cases. It revealed that an increased SOCS3 expression was associated with a better DFS and OS in both patients with HCC or other types of solid tumor cases (Figures 4C,D). These results were consistent with the above pooled analysis.

**Figure 5:**
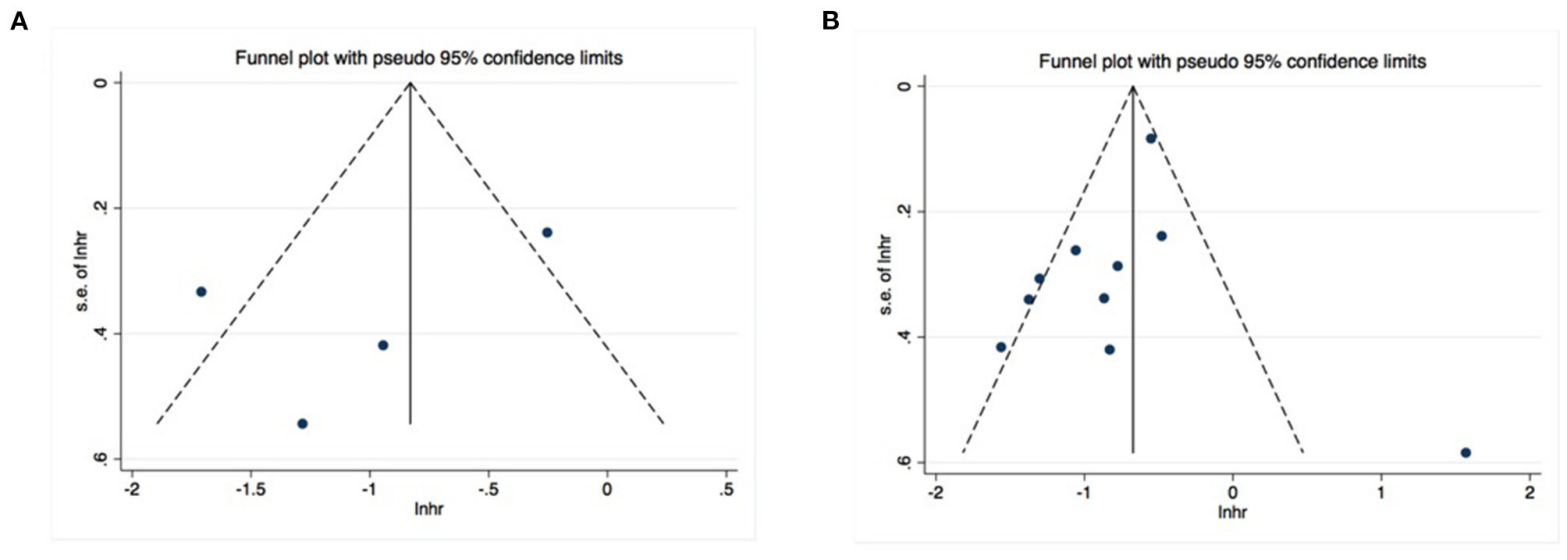
No significant publication bias existed in the pooled analysis about disease-free survival **(A)** or overall survival **(B)**.

Publication bias was investigated using Begg's and Egger's tests, as well as funnel plots. All *P*-values obtained from Egger's and Begg's tests for each endpoint were >0.05 (Table 2). Additionally, the visual inspection of the funnel plots did not show obvious asymmetry for the DFS (Figure 5A) or OS (Figure 5B) analyses. These results confirmed the absence of publication bias risk among the included studies in the present meta-analysis.

## Discussion

Due to the heterogeneity of cancer, the exploration of reliable prognostic biomarkers is highly important in the era of precision medicine. According to preclinical research studies, SOCS3 is a differently expressive gene between tumors and adjacent tissues (7, 23). However, its prognostic value in patients with cancer remains poorly understood. In the present study, we screened the available literature and performed a meta-analysis to assess the correlation between SOCS3 expression and the risk of cancer relapse and mortality. The findings demonstrated that higher SOCS3 expression in tumor tissues was associated with a significantly better DFS and OS compared with low expression. The results were stable without obvious publication bias. Meanwhile, we uncovered that SOCS3 was obviously related to Edmondson grading, vascular invasion, and metastasis instead of with tumor size and lymph nodes invasion. Generally, a few genes perform different effects on tumor biology or reflect inconsistent clinicopathological features. We believe the different results were mainly due to the limited sizes of available studies and different cancer types. According to these results, SOCS3 may be a novel prognostic biomarker in patients with solid tumors.

The exact mechanism through which SOCS3 affects patient outcomes remains unclear. Nevertheless, its inhibitory effect on multiple cytokine-related signaling pathways has been widely acknowledged. The incidence and progression of cancer may be ascribed to a variety of cytokines and growth factors, which are abundant in the tumor microenvironment (TME). In fact, a complex network of growth exists in TME and plays a critical role in cell-cell communication. The interaction of these cytokines with their receptors activates multiple signaling pathways, thus resulting in cell proliferation, angiogenesis, immune escape, and other biological processes which are hallmarks of cancer.

As mentioned above, members of the SOCS family of genes exert their anti-cancer function by inhibiting multiple signaling pathways related to cytokines and growth factors. In particular, SOCS3 mainly suppresses the activity of the interleukin 6/Janus kinase/signal transducer and the activator of the transcription 3 (IL6/JAK/STAT3) pathway in a feedback manner (24). It has been reported that SOCS3 is frequently silenced by hypermethylation and suppresses cell growth in human lung cancer (25). An *in vivo* study revealed that SOCS3 deficiency would induce gastric cancer by enhancing the STAT3 signaling pathway (26).

Cytokine IL6 is secreted by tumor cells or tumor stem cells and can promote cancer progression by mediating drug resistance, immune escape, angiogenesis, and metabolic disorder (27). Therefore, IL6 is considered an effective anti-cancer target. Accordingly, several monoclonal antibodies against IL6 or its receptor have been investigated in early-phase clinical trials for the treatment of hematological malignancy (28, 29), prostate cancer (30), renal cancer (31), and ovarian cancer (32). Targeting IL6 has shown a favorable safety profile and promising efficacy in the field of cancer management.

STAT3 is activated by JAK and IL6 and is a multi-function gene. Based on previous findings, STAT3 can facilitate cancer progression by increasing the expression of programmed cell death 1 ligand 1 (PD-L1), vascular epidermal growth factor A (VEGFA), matrix metalloproteinase (MMP), etc. (33). Importantly, STAT3 phosphorylation has been linked to worse and better prognosis in patients with solid tumors and hematological malignancy, respectively (34, 35). A previous meta-analysis confirmed that the overexpression of p-STAT3 was significantly correlated with poor outcomes in patients with cancer (36–38). Therefore, STAT3 is commonly considered a potential anti-cancer target (39). Recently, multiple promising STAT3 inhibitors have been used in clinical trials for the treatment of patients with cancer. Based on these data, we propose that the SOCS3-induced suppression of the IL6/JAK/STAT3 pathway may be one of the main mechanisms influencing patient outcomes (40).

To our knowledge, this is the first meta-analysis confirming the prognostic value of SOCS3 in patients with solid tumors. In clinical practice, SOCS3 could be recommended as a general molecular to be detected in tumor mass, which may assist physicians in recognizing high-risk patients and, consequently, achieve precision management. However, a well-designed prospective study is necessary to validate the prognostic value of SOCS3 in cancer patients.

However, some limitations in the present study should be acknowledged. Firstly, all included investigations were retrospective cohort studies with a modest level of evidence. Secondly, most participants in these studies were from Asian countries (e.g., China and Japan), which may restrict the applicability of these findings to populations residing in other regions. Thirdly, the heterogeneity detected in the present study may result from the quality of the studies included and multiple tumor types. Lastly, the cut-off value of SOCS3 expression in each study was inconsistent; this inconsistency may have affected our results.

## Conclusion

The anti-oncogene SOCS3 may be a novel biomarker for predicting the outcomes of patients with solid tumors. This molecule can be applied to clinical practice and may be a therapeutic target.

## Data Availability Statement

The original contributions presented in the study are included in the article/supplementary material. Further inquiries can be directed to the corresponding author.

## Author Contributions

JQ and YG: conception and design. XZ: data analysis and interpretation. JS and SW: collection and assembly of data. All authors wrote the manuscript and approved the final version of the manuscript.

## Funding

This study was funded by National Traditional Chinese Medicine Inheritance and Innovation Platform Construction Project by National Administration of Traditional Chinese Medicine, College Project of Jiangsu Province Hospital of Chinese Medicine (NO. Y2020CX570).

## Conflict of Interest

The authors declare that the research was conducted in the absence of any commercial or financial relationships that could be construed as a potential conflict of interest.

## Publisher's Note

All claims expressed in this article are solely those of the authors and do not necessarily represent those of their affiliated organizations, or those of the publisher, the editors and the reviewers. Any product that may be evaluated in this article, or claim that may be made by its manufacturer, is not guaranteed or endorsed by the publisher.
